# Computational modelling, green synthesis and biological activity of arylsulfonilamides as NNRTIs against HIV-1

**DOI:** 10.1186/1471-2334-14-S3-E4

**Published:** 2014-05-27

**Authors:** Anuradha Singh, Madhu Yadav, Nidhi Singh, Ritika Srivastava, Rajinder Kaur, Satish K Gupta, Ramendra K Singh

**Affiliations:** 1 Nucleic Acids and Antiviral Research Laboratory, Department of Chemistry, University of Allahabad, Allahabad- 211002, India; 2 National Institute of Immunology, Aruna Asaf Ali Marg, New Delhi, India

## Background

The development of new and potent anti-HIV compounds has become almost obligatory for the scientific community due to rapid emergence of drug resistant mutations. In pursuance of developing new anti-HIV molecules, we herein report the design, synthesis and anti-HIV properties of a series of potent arylsulfonilamide derivatives as NNRTIs.

## Methods

Development of arylsulfonilamides as NNRTIs involved both the computational and synthetic methods. On the basis of extensive docking experiments, ten promising compounds out of **55** initially taken were synthesized using green protocols and their *in vitro* anti-HIV activity assessed in TZM bl cells by luciferase assay and reverse transcriptase (RT) inhibition assay against wild type HIV-1 RT.

## Results

The compounds showed very promising *in silico* results as reflected by their lower ∆G values-high binding affinity, significant scoring functions high RT- ligand stabilization energy and close interatomic contacts through strong H-bonds with Lys103, His235, Tyr318, Lys101 and Val179; pi-pi interaction with Tyr181 and pi-cation interaction through Lys101, which all together predicted high EC_50_ values. However, the molecules showed unimpressive inhibitory action against HIV-1 under *in vitro* conditions. The encouraging part of this study was that these compounds behaved as NNRTIs as per our expectations, on the basis of results obtained during HIV-RT assay.

## Conclusions

In the present study, it has been observed that promising *in silico* results are not always corroborated by the desired *in vitro* results. Nevertheless, it is a part and parcel of drug discovery process, where successful drug development is nearly a hard nut to crack.

